# Effect of Ketoprofen and ATB-352 on the Immature Human Intestine: Identification of Responders and Non-responders

**DOI:** 10.1097/MPG.0000000000002308

**Published:** 2019-02-14

**Authors:** Marie-Pier Thibault, Éric Tremblay, John L. Wallace, Jean-François Beaulieu

**Affiliations:** ∗ Department of Anatomy and Cell Biology, Faculty of Medicine and Health Sciences, Université de Sherbrooke, Sherbrooke, Québec; † Department of Physiology and Pharmacology, University of Calgary, Calgary, Alberta, Canada.

**Keywords:** damaging effects, immature intestinal mucosa, nonsteroidal anti-inflammatory drugs, personalized response

## Abstract

**Background and Objective:**

The use of nonsteroidal anti-inflammatory drugs (NSAIDs) is associated with a broad spectrum of life-threatening adverse effects on the immature gastrointestinal tract. NSAID derivatives exploiting the beneficial effects of biologically active gases, such as hydrogen sulfide (H_2_S), have been developed. Herein, we determined the effects of ketoprofen and ATB-352, a H_2_S-releasing ketoprofen derivative, on selected metabolic pathways previously identified to be significantly altered by indomethacin in the human immature intestine.

**Methods:**

Ketoprofen and ATB-352 were tested on human mid-gestation small intestinal explants maintained in a serum-free organ culture system for 48 hours. The expression levels of the representative genes involved in selected metabolic pathways were measured by real-time PCR after a treatment of 48 hours.

**Results:**

Tested at a concentration that allows more than 80% inhibition of PGE2 production, ketoprofen was found to be less damaging than indomethacin at an equivalent dosage. However, based on the inducibility of cyclooxygenase-2 transcript expression, we were able to discriminate between responder individuals in which the deleterious effects observed with indomethacin were attenuated, and non-responder specimens in which the effects were similar to those observed with indomethacin. ATB-352 did not induce significant changes compared to ketoprofen on these metabolic pathways.

**Conclusions:**

These results show less damaging effects of ketoprofen compared to indomethacin on the immature intestine and indicate that the intestinal response to this NSAID significantly varies between individuals. However, the results did not allow us to demonstrate a specific beneficial effect of H_2_S release in organ culture.

**What Is Known**

- The administration of nonsteroidal anti-inflammatory drugs, such as indomethacin and ibuprofen, is associated with several adverse effects in neonates.
- Indomethacin impairs key metabolic pathways in the immature human intestine.

**What Is New**

- Ketoprofen generates fewer deleterious effects than other nonsteroidal anti-inflammatory drugs on the human mid-gestational intestine, possibly due to its role as double inhibitor of the enzymes involved in arachidonic acid metabolism.
- The intestinal response to at least some nonsteroidal anti-inflammatory drugs may vary from individual to individual.
- As compared to ketoprofen alone, an effect of the H_2_S-releasing derivative of ketoprofen could not be demonstrated in the organ culture set up.

Nonsteroidal anti-inflammatory drugs (NSAIDs) are among the most widely used drugs in the neonatal period. NSAIDs, such as indomethacin and ibuprofen, are used as tocolytic agents (1) as well as for the treatment of the most common cardiovascular abnormality among neonates, the patent ductus arteriosus (PDA) (2). It is well known that the administration of NSAIDs, in adults, is associated with several adverse effects, particularly gastrointestinal, such as gastropathies and enteropathies (3–6). It is therefore not surprising to see that those NSAIDs also cause several harmful side effects in neonates. In addition to being associated with a greater risk of miscarriage and congenital malformation, pre- and post-natal exposure to NSAIDs are also responsible for several disorders affecting many of the infant's organs, most notably the gastrointestinal tract (7). We have previously demonstrated that NSAIDs, such as indomethacin, can directly induce damaging effects on the human immature intestine (8).

A number of derivatives have been developed with the aim of minimizing the deleterious effects brought on by the administration of classic NSAIDs. Among these, some have exploited the beneficial cellular effects known for biologically active gases, such as hydrogen sulfide (H_2_S) (9). Indeed, several studies have shown that H_2_S could counterbalance some of the adverse effects brought on by the administration of NSAIDs by limiting certain elements of inflammation (10). The contribution of H_2_S occurs in 2 ways: by anti-inflammatory actions, such as the inhibition of the activation of NFκB, and by protective effects on the gastrointestinal tract, as the inhibition of leukocyte adhesion and the reduction of pro-inflammatory cytokine expression, such as TNFα, IL-1β and IFNγ (11–13). Recent studies have shown that the H_2_S-releasing derivatives are able to prevent the development of intestinal damage while being as effective as their classic counterparts (11,14,15). In this study, we tested another NSAID, ketoprofen and its H_2_S-releasing derivative on the immature small intestine in organ culture.

## METHODS

### Tissues

To evaluate the impact of ketoprofen and its H_2_S-releasing derivative ATB-352 on the human immature intestinal mucosa, a set of 6 specimens of small intestine (ileum) obtained from 6 fetuses following legal pregnancy interruption between 17 and 20 weeks of gestation were used. No tissues were collected from cases associated with known fetal abnormalities or intrauterine fetal demise. Studies were approved by the institutional review board for the use of human material from the “Centre Intégré Universitaire de Santé et de Services Sociaux de l’Estrie - Centre Hospitalier Universitaire de Sherbrooke.”

### Serum-free Organ Culture

Small intestinal tissues (ileum) obtained from 6 fetuses were prepared for organ culture as previously described (16,17). Briefly, each intestinal tissue was cut into several 5 × 5 mm explants which were maintained in organ culture dishes (Falcon Plastics, Fisher Scientific, Ottawa, ON, Canada) with Leibovitz L-15 serum-free culture medium containing 40 mg/mL amphotericin and 40 mg/mL mycostatin at the interface of a 95% air to 5% CO_2_ gas mixture at 37°C. Each culture dish contained 6 to 9 explants. Two dishes were used for each condition tested for each of the 6 small intestinal samples. Explants were maintained in culture for 48 hours and media was changed daily. The effects of ketoprofen and its derivative ATB-352 were tested at 1 and 10 μM. Prostaglandin E_2_ (PGE2) was measured as before (8).

### RNA Extraction, DNase Treatment, and Reverse Transcription

RNA was extracted with TRIzol (Invitrogen, Burlington, ON, Canada) according to the manufacturer's protocol and stored at –80°C. RNA samples first underwent DNase treatment (Invitrogen) according to the manufacturer's protocol and then reverse transcription of the samples was performed with Superscript II (Invitrogen).

### Real-time PCR

All reactions were performed in an Mx3000P real-time PCR system (Stratagene, Cedar Creek, TX) using Brilliant II SYBR Green QPCR Master Mix (Stratagene) in duplicate as previously described (18,19). Briefly, the run started with 5 minutes of Taq activation at 95°C followed by 40 cycles of melting (95°C, 30 seconds), primer annealing (55°C, 45 seconds) and extension (72°C, 45 seconds) ending with a melting curve analysis to validate the PCR product's specificity. Fluorescence data were acquired after each annealing step. The genes investigated in this study were ATP synthase, H+ transporting, mitochondrial Fo complex, subunit C1 (subunit 9) (*ATP5G1*), claudin-1 (*CLDN1*), cyclooxygenase-2 (*PTGS2*), chemokine, C-X-C motif, ligand 14 (*CXCL14*), cytochrome P450, family 3, subfamily A, polypeptide 4 (*CYP3A4*), dual oxidase 2 (*DUOX2*), intercellular adhesion molecule 1 (*ICAM-1*), NADH dehydrogenase (ubiquinone) 1 alpha subcomplex, 9, 39 kDa (*NDUFA9*), nitric oxide synthase 2 (*NOS2*), occludin (*OCLN*), superoxide dismutase 2 (*SOD2*), and trefoil factor 1 (*TFF1*). Peptidylprolyl isomerase A (*PPIA*) and ribosomal protein S23 (*RPS23*) were used as reference genes. Primers are listed in Supplemental Table 1 (Supplemental Digital Content) and were generated by the primer formation software Primer3 (*http://bioinfo.ut.ee/primer3*).

### Data Expression

The differential expression of genes was estimated by comparing the expression of untreated (control) and treated explants (ketoprofen or ATB-352) using the equation R = (E_target_)^ΔCttarget^/(E_reference_)^ΔCtreference^(20). Samples were normalized to a set of 2 reference genes, *PPIA* and *RPS23*. The ratio of ATB/ketoprofen was used to evaluate the additional effect of H_2_S on the human mid-gestational intestine compared with ketoprofen alone. The statistical analyses were made by the software GraphPad Prism 7 (GraphPad, San Diego, CA). The Wilcoxon test (when n = 6) and the one sample *t* test (when n = 3) were used to estimate the significance of the expression of a gene compared with its expression control (fixed to 1), while the Mann-Whitney test was used to compare the expression of a gene between the various treatments. *P* values lower than 0.05 (0.06 where specifically indicated) were considered significant.

## RESULTS

### Direct Effects of Ketoprofen on the Human Immature Intestinal Mucosa

**FIGURE 1:**
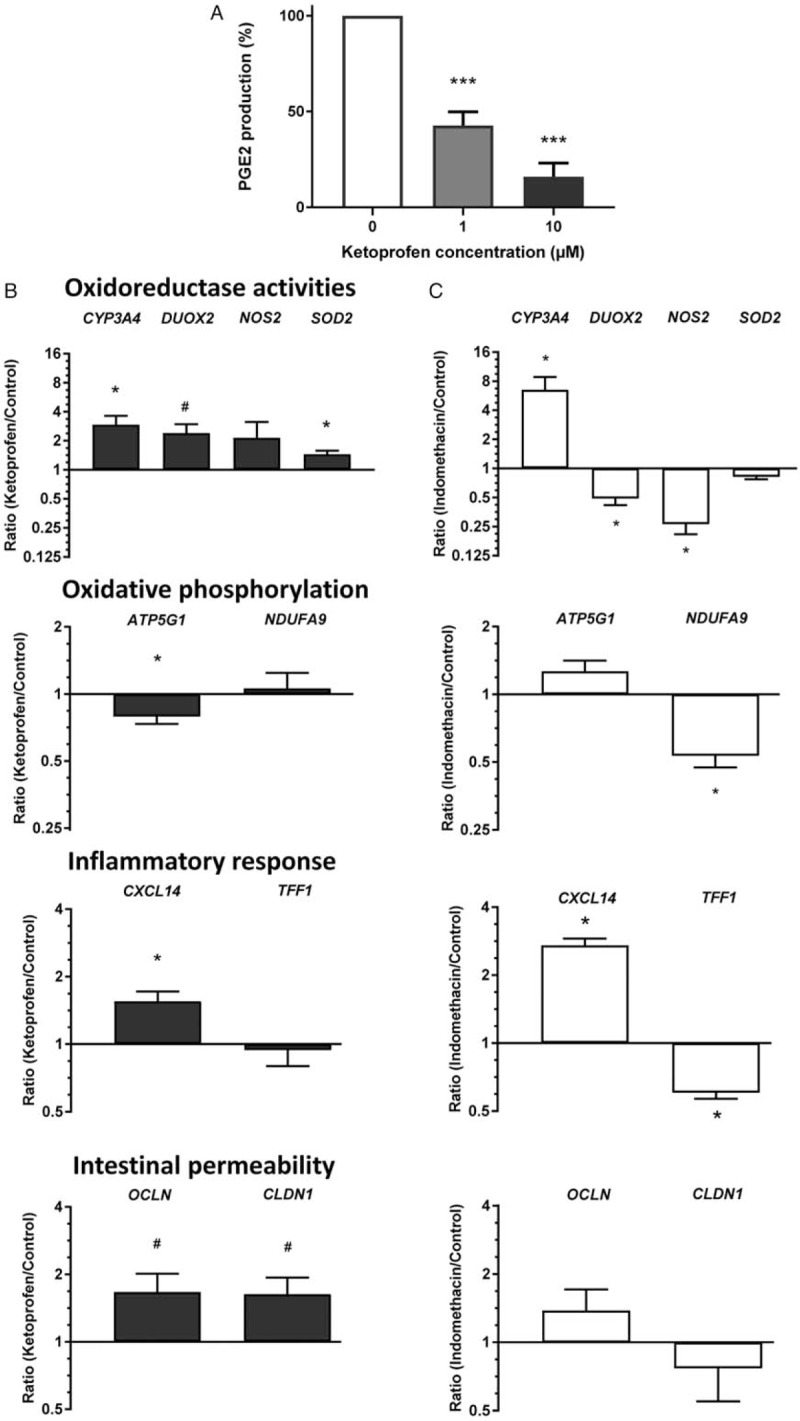
Effects of ketoprofen on the immature intestinal mucosa. A, Inhibition of PGE2 production was evaluated in the presence of 2 concentrations of ketoprofen (mean ± SEM, ^∗∗∗^*P* < 0.005). B, qPCR analysis of transcript levels for representative markers of mucosal homeostasis categories after 48 hours of culture in the presence of 10 μM ketoprofen. C, For comparison, markers expressed relative to the same housekeeping genes in response to 1 μM indomethacin under similar conditions. qPCR data are expressed as ratios of treated over untreated segments (Log2 scale). Values from 6 independent biological samples (mean ± SEM). ^∗^*P* < 0.05 (^#^*P* < 0.06) versus corresponding untreated control segments.

As previously validated for indomethacin testing, 1 approach to estimate relevant concentrations for testing NSAIDs on small intestinal explants is to mimic circulating levels reported to be efficient for patent ductus arteriosus closure in the neonate and then validate it for the inhibition of PGE2 production (8). Unfortunately, such data were not available for ketoprofen but in 1 study where this NSAID was used as a tocolytic agent, ketoprofen concentrations estimated to be between 2 and 12 μM were measured in the plasma of neonates in the first hours of life (21). When tested on small intestinal explants, the 1 and 10 μM concentrations were found to be sufficient to inhibit 60% and 80% of PGE2 production over a 48-hour period, respectively (Fig. 1A). Experiments were thus performed with the 2 concentrations but only data with 10 μM were further considered.

To investigate the influence of ketoprofen on the mid-gestation intestine, matched control and ketoprofen-treated explants were tested for the expression of markers for specific metabolic pathways previously identified as being significantly altered in response to indomethacin (8,19). Ketoprofen treatment was found to increase expression of *CYP3A4*, *DUOX2*, and *SOD2*, 3 markers of oxidoreductase activity, CXCL14, a marker of the inflammatory response, and OCLN and CLDN1, 2 structural components of the tight junctions while a reduction of the expression of ATP5G1, a marker of the oxidative phosphorylation pathway, was observed (Fig. 1B). Unexpectedly, these results were found to be different to those observed with indomethacin at comparable effective concentrations (8,19). To allow a more direct comparison, our previous data with indomethacin were recalculated using the same new set of housekeeping genes (*PPIA* and *RPS23*) chosen on the basis of their stability in intestinal tissue under stressful conditions (Thibault MP, unpublished data). Recalculation with the new reference genes had little influence on the overall results as shown in Fig. 1C, where only 2 of the 10 tested genes, *CYP3A4* and *CXCL14*, appear to follow the same trend in response to ketoprofen and indomethacin treatments, confirming the differential intestinal response between these 2 NSAIDs.

### Two Patterns of Response: Ketoprofen Responders and Non-responders

**FIGURE 2:**
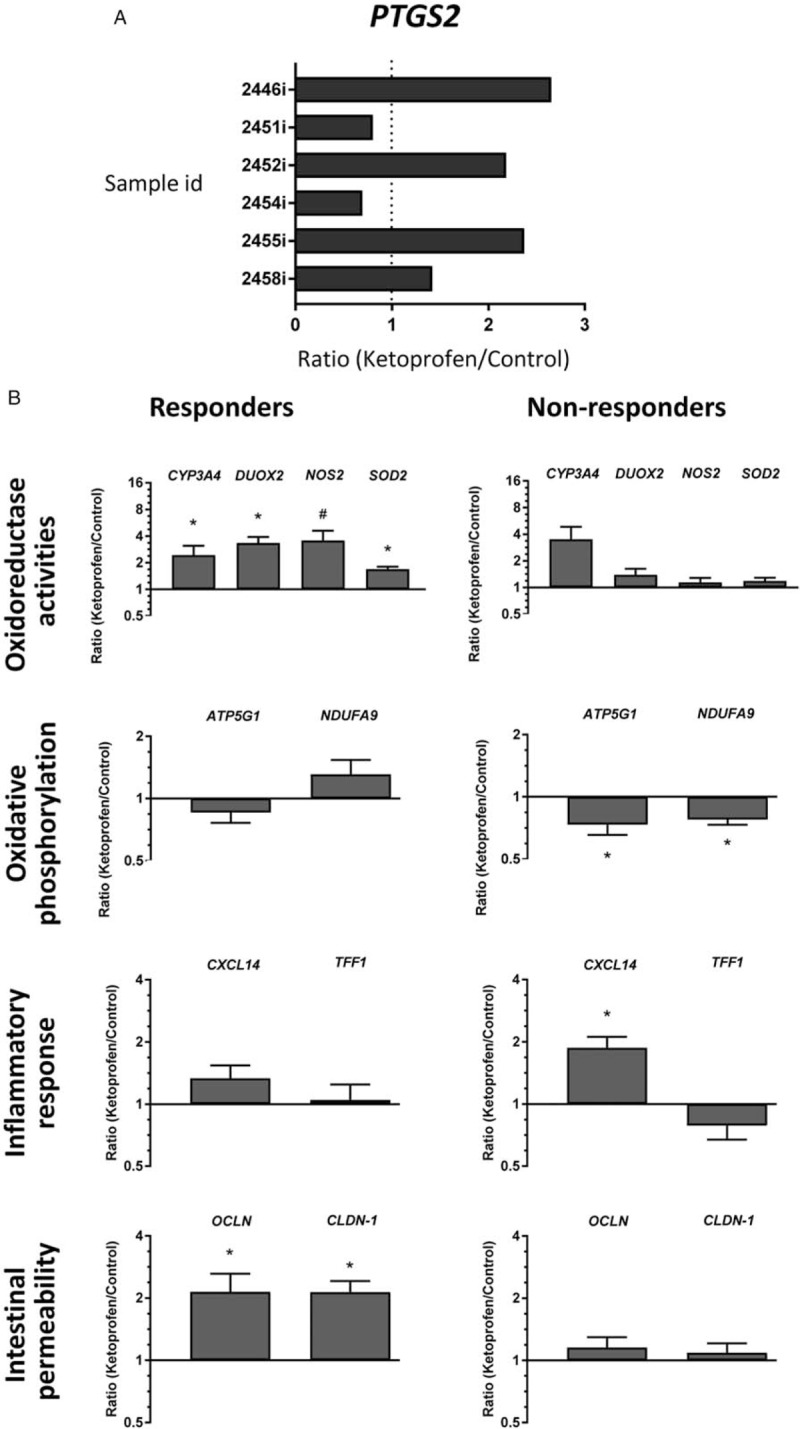
Characterization of intestinal ketoprofen responders and non-responders. A, qPCR analysis of PTGS2 transcript levels in the 6 independent explant cultures. B, Based on data from (A) qPCR data for the markers of mucosal homeostasis are shown for the 3 responders and the 3 non-responders to ketoprofen. qPCR data are expressed as ratios of treated over untreated segments for each sample for (A) and for the mean of 3 independent biological samples for each responder group (Log2 scale, ^∗^*P* < 0.05, ^#^*P* < 0.06) versus corresponding untreated control segments (mean ± SEM).

Surprisingly, analysis of *PTGS2* (cyclooxygenase-2) in ketoprofen-treated explants revealed highly variable levels of expression of this gene from sample to sample. In fact, as shown on Figure 2A for 6 independent cultures, 3 were found to be unaltered (<50% variation; 0.97 ± 0.23, NS) while the 3 others were induced by more than 100% (2.40 ± 0.14, *P* = 0.0006) suggesting that some samples were “responders” to ketoprofen treatment at the transcript level while others were “non-responders.” Incidentally, *PTGS2* analysis from the indomethacin treated samples showed that the responder/non-responder phenomenon was not elicited, PTGS2 expression being uninduced by indomethacin (0.68 ± 0.18, NS).

We thus reanalyzed the data taking into consideration this distinction. In this context, overall the responders showed fewer harmful effects that the non-responders (Fig. 2B). As expected, non-responders displayed a deleterious indomethacin-like phenotype (compare Fig. 2B with Fig. 1C). Indeed, while the expression of *CYP3A4* tends to always increase, the expression of the other oxidoreductase activities tested varied largely between them, *DUOX2*, *NOS2*, and *SOD2* being upregulated only in the responders (Fig. 2B). Similarly, the level of genes involved in oxidative phosphorylation and inflammatory response was not altered in the responders while the non-responders displayed an indomethacin-like response. Finally, expression of the intestinal permeability markers *OCLN* and *CLDN1* increased in the responders but not in the non-responders (Fig. 2B).

### Impact of the Contribution of H_2_S by the Derivative ATB-352 on Gene Expression in the Human Immature Intestinal Mucosa

**FIGURE 3:**
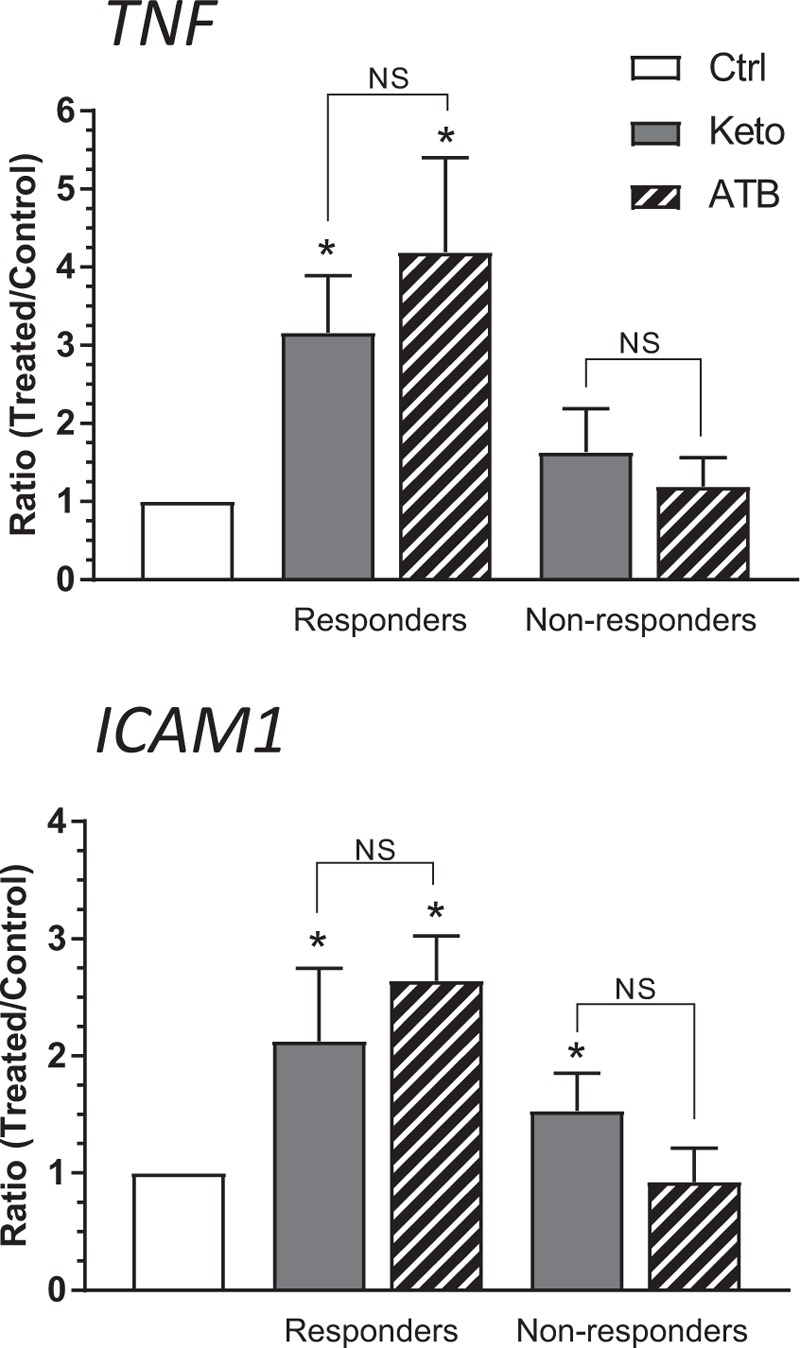
Analysis of expression of representative H_2_S-targeted genes in ATB-352 or ketoprofen treated intestine. qPCR analysis of TNFα and ICAM1 transcript levels in control (Ctrl), ATB-352 (ATB) or ketoprofen (Keto) treated responder and non-responder explants. Data are expressed as ratios of treated over control explants as the mean of 3 independent biological samples for each group (mean ± SEM). ^∗^Denotes a difference (*P* < 0.05) versus corresponding untreated control explants. NS = non-significant.

The evaluation of the effects of H_2_S on the human immature intestinal mucosa was made via the treatment of explants maintained in organ culture with ATB-352, a H_2_S-releasing derivative of ketoprofen. The qPCR analysis allowed the determination of the effects of H_2_S release on the metabolic pathways previously analyzed with ketoprofen alone. This comparison was made using an ATB/ketoprofen ratio to estimate the additive effects of a potential release of H_2_S on the human immature intestinal mucosa. In addition to some minor exceptions, the ATB compound did not seem to significantly affect the expression of the selected genes compared to ketoprofen alone (Table 1). Analysis of representative genes for which expression was found to be attenuated by H_2_S donors such as *ICAM-1* and *TNF*(12) confirmed the lack of effect of ATB in organ culture (Fig. 3).

## DISCUSSION

The anti-inflammatory properties of ketoprofen, like other classical NSAIDs, pass through the nonspecific inhibition of the isoenzymes COX-1 and COX-2 responsible for triggering the inflammation cascade via the production of prostaglandins. This study was undertaken with the aim of estimating the direct effects of ketoprofen on the human immature intestinal mucosa to help in the understanding of the molecular mechanism responsible for NSAID enteropathies (3,7) and to evaluate the potential beneficial effects of exposure to H_2_S using ATB-352, a H_2_S-releasing derivative of ketoprofen (14). Overall, while our data showed a relative inefficiency of H_2_S release on the immature small intestine in the organ culture setup, it revealed a previously undisclosed phenomenon pertaining to a distinct intestinal response to ketoprofen among individuals referred to above as responders versus non-responders.

The initial analysis of the effects of ketoprofen, which included 6 independent cultures, showed some trends in the modulation of pro-inflammatory-related genes such as an increase of *CXCL14* expression, a pro-inflammatory cytokine (22). Increase of *CYP3A4*, an abundant form of the cytochrome P450 in the intestine responsible for the metabolism of drugs (23), was also observed but the overall response to ketoprofen was found to be quite distinct to what had been previously reported by our group under similar conditions with another NSAID, indomethacin (8). A more detailed analysis of the data revealed however that in contrast to COX activity, which was consistently inhibited by ketoprofen (as evaluated by PGE2 production), COX2 mRNA (*PTGS2*) expression levels were highly variable between the independent cultures. Incidentally, as for *PTGS2*, a large proportion of the tested markers used for monitoring the inflammatory response also displayed large standard deviations. Further analysis of the data revealed that the intestine of the 3 individuals showing a significant induction in the expression of *PTGS2* were also exhibiting a typical protective anti-inflammatory gene expression profile similar to that reported by our group after epidermal growth factor treatment with increase in levels of anti-oxidative stress markers such as DUOX2, SOD2, NOS2 and the tight junction components CLDN1 and OCN (18,19). The intestines of these individuals were thus referred to as the “responders.” In contrast, in the intestine of the 3 non-responders, *PTGS2* expression was not modulated and the tissues displayed a deleterious pro-inflammatory response gene expression signature similar to the one observed with indomethacin (8). It is noteworthy that this responder/non-responder phenomenon was not observed with indomethacin since, based on the lack of *PTGS2* induction at the transcript level, all samples behaved as non-responders.

A possible explanation for such a distinct mucosal response to ketoprofen versus indomethacin relies on a recent finding suggesting distinct mechanisms of action for these 2 NSAIDs. Indeed, as for the majority of NSAIDs, indomethacin exerts its anti-inflammatory properties by inhibiting the main arachidonic acid metabolism pathway, namely the production of prostaglandins, through the inhibition of COX activities (6,24,25). However, the inhibition of prostaglandin synthesis by indomethacin leads to higher arachidonic acid metabolism by the alternative 5-lipoxygenase (5-LO) pathway responsible for leukotriene production, including leukotriene B4 (LTB4), an important inflammatory mediator (6,24,26,27). In addition to increasing microvascular permeability and promoting neutrophil infiltration, LTB4 also stimulates the production of reactive oxygen species by the release of superoxide by neutrophils and hydrogen peroxide by macrophages (28,29) suggesting a potential source of inflammatory-related damage observed by indomethacin on the immature intestine. In contrast, ketoprofen appears to act as a dual COX and 5-LO inhibitor, blocking the synthesis of prostaglandins and leukotrienes (30), thus providing the basis for a possible explanation for the observed divergence between the 2 treatments on the immature intestine at least for a subset of individuals. Further work is nevertheless needed to better understand the mechanisms regulating this intestinal responder/non-responder phenomenon. No correlation with the sex of the individual or gestational age can explain the phenomenon. However, genetic susceptibilities associated to NSAID metabolism have been identified in relation to gastrointestinal bleeding (31), while identification of genetic variants in lipoxygenases and cyclooxygenases have been reported in association with the risk of digestive neoplasia (32–34) suggesting that the implication of such polymorphisms in the differential intestinal response to ketoprofen cannot be ruled out at this time.

Finally, the testing of ATB-352, a ketoprofen derivative bound to a 4-hydroxythiobenzamide group which allows the release of H_2_S (15), in an organ culture system enabled us to evaluate the direct effects of the compound on the immature intestinal mucosa. In vivo, several studies have shown that the administration of a H_2_S donor prevents the oxidative damage caused to the intestinal mucosa in addition to limiting certain elements of inflammation brought on by the administration of NSAIDs (11,13–15). For instance, H_2_S can inhibit leukocyte adhesion and migration to the sites of inflammation in addition to reducing the production of pro-inflammatory cytokines through a mechanism that appears to involve suppression of nuclear factor kB activity (12,15). Our data indicate that ATB-352 has similar effects as ketoprofen on the immature intestinal mucosa (including the responder/non-responder-type response) suggesting that the H_2_S release occurs rapidly in an open in vitro system. Incidentally, H_2_S is known to be released within minutes after administration in vivo (15). The limited influence on mucosal cells under these conditions was confirmed by the analysis of *ICAM-1* and *TNF*, 2 specific H_2_S target genes (12). As recently suggested for nitric oxide, another potential protective molecule for the intestinal mucosa (19), future studies could include an adaptation of the organ culture system for analyzing the direct effects of specific gaseous forms such as H_2_S on the intestinal mucosa.

Taken together, these results show that ketoprofen induces fewer deleterious effects than the NSAID indomethacin on the immature small intestinal mucosa and that the difference is the result of a distinct individual intestinal response to ketoprofen, half of the samples exhibiting a protective response that included upregulation of anti-oxidative stress markers and tight junction components while the other half displayed a deleterious response comparable to the one observed with indomethacin. Future studies with a larger cohort should help to further document this phenomenon. The facts that in contrast to most other NSAIDs ketoprofen has been reported to inhibit both the COX and 5-LO pathways and the existence of polymorphisms susceptible to affecting these pathways are 2 aspects that would need to be considered.

## Supplementary Material

Supplemental Digital Content

## Figures and Tables

**TABLE 1:** qPCR analysis of the effects of H_2_S released on the expression of representative markers for distinct mucosal homeostasis categories in ketoprofen responders and non-responders

	Responders		Non-responders	
Genes	Ratio ATB/Keto^*^	*P*	Ratio ATB/Keto^*^	*P*
Oxidoreductase activity				
*CYP3A4*	1.00 ± 0.44	0.9907	2.07 ± 2.19	0.4866
*DUOX2*	1.61 ± 0.34	0.0900	0.43 ± 0.46	0.1616
*NOS2*	1.60 ± 0.27	0.0596	0.33 ± 0.24	0.0418^†^
*SOD2*	1.22 ± 0.16	0.1374	0.78 ± 0.29	0.3251
Oxidative phosphorylation				
*ATP5G1*	1.24 ± 0.40	0.4064	0.83 ± 0.10	0.0909
*NDUFA9*	1.21 ± 0.41	0.4644	0.93 ± 0.44	0.8155
Inflammatory response				
*CXCL14*	1.38 ± 0.07	0.0125^†^	1.21 ± 0.24	0.2636
*TFF1*	1.26 ± 0.13	0.0724	0.85 ± 0.53	0.6710
Permeability				
*CLDN-1*	1.13 ± 0.44	0.6663	0.73 ± 0.17	0.1134
*OCLN*	1.27 ± 0.61	0.5276	0.91 ± 0.43	0.7609

^*^Data are expressed as ratios of ATB-352 over ketoprofen (ATB/Keto)-treated immature intestinal explants in both ketoprofen responder and non-responder groups. Data are expressed as the mean ratio of 3 independent biological samples for each group.

^†^Denote a statistical difference.
