# Supplementary material for: Effect of Ketoprofen and ATB-352 on the Immature Human Intestine: Identification of Responders and Non-responders
Source: J Pediatr Gastroenterol Nutr. 2019 Feb 14;68(5):623–9. doi: 10.1097/MPG.0000000000002308 (PMC6510328; doi:10.1097/MPG.0000000000002308)
Supplement: Supplemental Digital Content [file jpga-68-623-s001.docx]

**Supplemental Table 1**. Primers used in this study

| Gene symbol | Sense primer | Antisense primer | Accession no. |
| --- | --- | --- | --- |
|  |  |  |  |
| ATP5G1 | 5’-ACAGCAACTTCCCACTCCAG-3’ | 5’-GCCAAGAATGGCATAGGAGA-3’ | NM_005624 |
| CLDN1 | 5’-CCGTTGGCATGAAGTGTATG-3’ | 5’-CCAGTGAAGAGAGCCTGACC-3’ | NM_021101 |
| CXCL14 | 5’-AAGCTGGAAATGAAGCCAAA-3’ | 5’-GGCGTTGTACCACTTGATGA-3’ | NM_004887 |
| CYP3A4 | 5’-CAAGACCCCTTTFTFFAAAA-3’ | 5’-CGAGGCGACTTTCTTTCATC-3’ | NM_017460 |
| DUOX2 | 5’-CGGCAATCATCTATGGAGGT-3’ | 5’-ATGTGCAGGCTGAGTGTGTC-3’ | NM_014080 |
| ICAM-1 | 5’-CTTGAGGGCACCTACCTCTG-3’ | 5’-CATTATGACTGCGGCTGCTA-3’ | NM_000201 |
| NDUFA9 | 5’-CCGGAAGCCATTATCGTAAA-3’ | 5’-TTCACCAGGTGGAAAAGGAG-3’ | NM_005002 |
| NOS2 | 5’-CTCTATGTTTGCGGGGATGT-3’ | 5’-TTCTTCGCCTCGTAAGGAAA-3’ | NM_000625 |
| OCLN | 5’-TCCAATGGCAAAGTGAATGA-3’ | 5’-GCAGGTGCTCTTTTTGAAGG-3’ | NM_002538 |
| PPIA | 5’-GGCAAATGCTGGACCCAACACA-3’ | 5’-TGCTGGTCTTGCCATTCCTGGA-3’ | NM_021130 |
| PTGS2  RPS23 | 5’-ATCACAGGCTTCCATTGACC-3’  5’-AGGAAGTGTGTAAGGGTCCAGC-3’ | 5’-CAGGATCACGCTCCACAGCA-3’  5’-CACCAACAGCATGACCTTTGCG-3’ | NM_000963  NM_001025 |
| SOD2 | 5’-GGAAGCCATCAAACGTGACT-3’ | 5’-CTGATTTGGACAAGCAGCAA-3’ | NM_001024465 |
| TFF1 | 5’-CACCATGGAGAACAAGGTGA-3’ | 5’-TGACACCAGGAAAACCACAA-3’ | NM_003225 |
